# Dreams Realized: A Long-Term Program Evaluation of Three Summer Diversity Pipeline Programs

**DOI:** 10.1089/heq.2020.0126

**Published:** 2021-08-04

**Authors:** Cara Stephenson-Hunter, A. Hal Strelnick, Natalia Rodriguez, Luciana A. Stumpf, Hope Spano, Cristina M. Gonzalez

**Affiliations:** ^1^ Department of Family and Social Medicine, Albert Einstein College of Medicine, Bronx, New York, USA.; ^2^ Hispanic Center of Excellence's Summer Undergraduate Program, Albert Einstein College of Medicine, Bronx, New York, USA.; ^3^ Department of Medicine, Albert Einstein College of Medicine, Bronx, New York, USA.

**Keywords:** pipeline programs, health workforce, diversity programs, URM students, social cognitive theory, premedical students

## Abstract

**Purpose:** Pipeline programs are a well-known approach to enhancing health care workforce diversity and reducing health disparities. Few evaluations of pipeline programs include long-term outcome; fewer still, if any explore perceptions of students after completing such programs, to elucidate factors that contribute to successful entry into the health professions. The authors conducted a program evaluation of three summer diversity pipeline programs in the Bronx, NY, investigating both long-term outcomes and participants' hindsight perspectives of the impact of these programs on their career trajectories.

**Methods:** Investigators conducted a cross-sectional, long-term, mixed-methods survey study. The primary and secondary outcomes for the quantitative analysis were matriculation into biomedical programs to pursue MD or PhD degrees and Master's degrees, respectively, and associated demographic factors. Free-text questions explored the most valuable and influential components of the programs; responses were analyzed qualitatively.

**Results:** Of 147 respondents, 107 (73%) were on-track or had entered a doctoral or master's program, achieving either the primary or secondary outcomes, respectively. Components cited as most valuable included clinical experience, mentorship, career exposure, and research opportunities. Three themes were identified from the free-text responses: (1) Dreams realized; (2) Professional identity formation; and (3) Addressing systemic inequities.

**Conclusions:** These three pipeline programs achieved career outcomes similar to published data. Participants' insights highlight the value of relationships, direct exposure to the health professions, and the importance of such programs to address systemic barriers faced. Results can inform criteria both for participant selection, as well as benchmarks used to define individual and programmatic success.

## Introduction

I come from a low-income family and will be the first to graduate from professional school and college. I would have never had the exposure and mentorship that has been essential in my success. This program changed my life.

A more diverse health professions workforce contributes to reducing racial and ethnic health disparities.^1–4^ Yet, the diversity within the health professions is not increasing at a sufficient rate to reflect the racial, ethnic, and linguistic diversity of the current or future U.S. population.^3,5^ The Association of American Medical Colleges (AAMC) defines underrepresented minorities (URM) as “racial and ethnic populations that are underrepresented in the medical profession relative to their numbers in the general population.”^6^ While URMs represent 29% of the total U.S. population, the percentage of URMs entering medical school in 2018–2019 was only 13% (7.1% African American, 6.2% Latino, and 0.2% American Indian/Alaska Native).^7^ In response, many institutions offer “pipeline” programs, interventions addressing the educational and social barriers to successful matriculation into health professional schools faced by economically and educationally underserved and/or URM groups.^8–12^ Programs seek to “increase the number of well-prepared URM students (in grades K-16) motivated to enter the health professions.”^2^

To achieve their goals, pipeline programs use a variety of intervention models and approaches to academic and professional enrichment and support. Limited program evaluation data hinder program developers' ability to quantify successful attainment of stated goals: A consensus on the methods and benchmarks used to define successful programs and the factors that influence them are lacking.^8^ Few program evaluations describe the impact of these programs beyond short-term outcomes.^10,13,14^ This gap in our knowledge is further compounded by minimal reports about the personal impact they have on participants, and what elements contribute most to the students' perception of their own success pursuing a health career.^15–17^ Without a better understanding of the components of pipeline programs that most effectively contribute to participant success, we risk wasting opportunities and resources, an especially important risk to avoid given the current, limited funding climate.^2^ The federal Health Careers Opportunity Program, the major funding source for pipeline programs since 1972, has for more than a decade received less than half its FY2005 level.^18^

To address this relatively underexplored topic, we conducted a long-term follow-up study of participants in three summer diversity pipeline programs. The objectives of this study were to (1) investigate participants' long-term educational and career outcomes and (2) explore participants' perspectives of the impact of the programs on their academic and career trajectories.

## Methods

### Program descriptions

The Albert Einstein College of Medicine and its university hospital, Montefiore Medical Center, are located in Bronx, NY. Its three pipeline programs are the Summer Undergraduate Mentorship Program (SUMP), Montefiore Health Opportunities Program (Monte-HOP), and Diversity Student Summer Research Opportunity Program (DSSROP). Although the three summer pipeline programs differ in their selection criteria and intervention approaches, they share an overall goal of diversifying the health care workforce. A detailed side-by-side comparison of the programs is presented in Table 1.

**Table 1.** Descriptions of Summer Enrichment Pipeline Programs Conducted at the Albert Einstein College of Medicine/Montefiore Medical Center, Bronx, NY

	SUMP	Monte-HOP	DSSROP
Mission/goals	• To expose students to careers in health professions • To enhance students' ability to apply and matriculate into health professional school	• To promote, educate, and encourage youth from underserved backgrounds to pursue careers within the health fields	• To provide students educational opportunities for students interested in a research career in the biomedical sciences
Participant eligibility	• Rising college sophomores through seniors • Economically or educationally disadvantaged backgrounds and/or from underrepresented groups in medicine • US Citizens or Permanent Residents	• Graduating high school seniors or rising college freshmen to juniors • Economically or educationally disadvantaged and/or from underrepresented groups in health care • US Citizens or Permanent Residents	• Rising college juniors and seniors • Underrepresented groups in medicine • US Citizens or Permanent Residents
Time commitment	• 6 weeks • 30 h/week	• 6 weeks • 30 h/week	• 8 weeks • 40 h/week
Didactic curriculum	• Clinical didactic • Medical informatics • Test taking strategies	• Study skills • Research skills • Clinical didactic	• Research skills • Academic, career, and financial seminars
Experiential curriculum	• Shadowing in clinical setting	• Shadowing in clinical setting	• Laboratory-based experience • (No shadowing in clinical setting) •
Structured mentorship	• Clinical mentor • Research mentor • Weekly “rap sessions”	• Clinical mentor	• Research supervisor •
Post-program follow-up	• Annual reunions • Second research summer • Contact with program administrator	• Annual reunions Second research summer (Alumni Research in Community Health & Equity Studies)	• None
Research	• Literature review based on clinical experiences • Formal oral presentation • Formal research paper	• Literature research project (library-based) • Formal oral presentation	• Formal laboratory research project • Formal poster presentation • Formal research paper
Parental involvement	• Parent workshops on financing medical education and medical school application process •	• Parent workshop on career opportunities for students in health professions	• None
Application process	• Online • Minimum GPA=2.5 • Essay • Letter of Recommendation (1) • Official transcript • Resume	• Paper (mailed in) • No minimum GPA • Essay • Letters of Recommendation (2) • Official transcript • Resume • Interview	• Paper (mailed in) • Minimum science GPA=3.0 • Essay • Letter of Recommendation (1) • Official transcript • Intent to pursue an MD or MD/PhD
Stipend	• $1000 upon completion • Travel vouchers (Metrocards)	• Varies by year of funding • Travel vouchers (Metrocards)	• $3000 (two installments of $1500)
Housing	• No	• No	• Yes
Funding sources	• HRSA (COE and HCOP)	• HRSA (MCH and HCOP) and institutional	• Institutional and NIH

DSSROP, Diversity Student Summer Research Opportunity Program; COE, Centers of Excellence; GPA, grade point average; HCOP, Health Career Opportunities Program; HRSA, Health Resources & Services Administration; MCH, Maternal Child Health; Monte-HOP, Montefiore Health Opportunities Program; SUMP, Summer Undergraduate Mentorship Program.

### Program evaluation

Social cognitive career theory (SCCT) served as our conceptual framework; it informed our survey design aiming to understand how students' experiences in the programs, including mentorship and exposure to clinical and research environments, influenced their academic and career choices.^19^ SCCT explains how learning experiences influence the development of education and career choice and achievement through cognitive factors, including self-efficacy and outcome expectations. The model been used for over 25 years, across numerous career settings, and within various underserved populations to explain career development.^20,21^

We conducted a cross-sectional mixed-method long-term program evaluation. Two authors (C.M.G. and H.S.) developed an 87-item electronic survey based on a literature review and on our previous experience administering pipeline programs. There was skip logic built into the survey, therefore individual respondents answered many fewer than 87 questions. Survey items included demographic information, academic and career achievements, most beneficial components of the program, and barriers to meeting career goals; it included three questions with the opportunity to provide free-text, narrative responses. All individuals who completed any of the three pipeline programs between the years of 2002–2012 and for whom we had contact information were invited to complete the confidential survey via email; three follow-up reminders were sent each spaced 2 weeks apart during the spring of 2013.

For the quantitative portion of our program evaluation, the primary outcome was matriculation into medical school or graduate school to pursue an MD or a PhD in the biomedical sciences. The secondary outcome was matriculation into a health professions school at the master's degree level. An age-neutral outcome of “on-track” was defined as progress toward the completion of the primary and secondary outcomes to assess the trajectories of younger students continuing or having recently completed their undergraduate education and recent graduates taking “gap” years. The study received exempt status by the Institutional Review Board of the Albert Einstein College of Medicine.

### Data analysis

Statistical analysis included descriptive statistics, including means and frequencies, of the percentage of total participants who achieved the primary or secondary outcome. Pearson's correlation and chi-square tests were conducted to identify any association between demographic variables, including sex, race/ethnicity, and academic level at the time of participation in the pipeline program, and study outcomes. A *p*-value <0.05 was considered statistically significant. Analyses were conducted using SPSS v.24.

Responses to the first open-ended question, “Please describe what was the most valuable to you during your pipeline experience,” were analyzed by frequency coding.^22^ Narrative responses to the remaining two open-ended questions were analyzed through thematic analysis.^23^ For the latter, three investigators (C.M.G., N.R., and L.S.) independently read one-third of the free-text responses line-by-line and applied categories following inductive coding to the text.^22^ They then met to discuss and refine their individual codes and devise an agreed upon list of codes along with their definitions to create the preliminary codebook. This codebook was independently applied by at least two investigators to the remaining free-text responses and further refined. Investigators then met to perform further analysis using a constant comparative method, a technique in which data are transformed into larger theoretical categories. Investigators began with low inference codes and discussed their meaning and potential grouping of codes to develop conceptual themes.^22^ Finally, the relationships between themes were identified, and consensus on representative quotes was reached by the three investigators.

## Results

Of our 461 summer program graduates, there were 361 with known addresses. We reached out to these 361 and received 158 responses to the survey, resulting in a response rate of 44% (158/361). Surveys without a response to the question, “Education completed since participating in last pipeline program” and duplicate surveys were excluded from our analysis, resulting 147 complete, distinct surveys. Demographic information describing our students is summarized in Table 2. No associations between demographic variables and primary or secondary outcomes reached statistical significance (*p*>0.05 for comparisons).

**Table 2.** Characteristics of Study Population by Program

Characteristics	SUMP respondents (N=84)	Monte HOP respondents (N=34)	DSSROP respondents (N=29)	Total respondents (N=147)
Age at time of survey completion				
Mean (SD)	23.38 (2.93)	22.97 (3.16)	23.91 (2.37)	23.38 (2.89)
18 and under, *n* (%)	1 (1)	2 (6)	0 (0)	3 (2)
19–21, *n* (%)	18 (21)	10 (29)	2 (7)	30 (20)
22–24, *n* (%)	24 (29)	7 (21)	12 (41)	43 (29)
25 and over, *n* (%)	23 (27)	12 (35)	9 (31)	44 (30)
Missing, *n* (%)	18 (21)	3 (9)	6 (21)	27 (18)
Self-identified race/ethnicity, *n* (%)				
Hispanic/Latino	53 (63)	14 (41)	12 (41)	79 (54)
Black (non-Hispanic/Latino)	8 (10)	12 (35)	10 (35)	30 (20)
Asian (non-Hispanic/Latino)	4 (5)	3 (9)	0 (0)	7 (5)
White (non-Hispanic/Latino)	0 (0)	0 (0)	0 (0)	
Mixed race (non-Hispanic/Latino)	1 (1)	1 (3)	1 (3)	3 (2)
Undisclosed race/ethnicity	18 (21)	4 (12)	6 (21)	28 (19)
Gender, *n* (%)				
Female	43 (51)	23 (68)	11 (38)	77 (53)
Male	23 (27)	8 (24)	12 (41)	43 (29)
Missing	18 (21)	3 (8)	6 (21)	27 (18)

SD, standard deviation.

Primary and secondary outcome achievements by program are listed in Table 3. Of the 34 who achieved our primary outcome, 65% were enrolled in medical school, 24% achieved medical school degrees, and 12% were enrolled in a biomedical doctoral program. For our on-track/off track outcome, 147 respondents, 107 (73%) were on-track. On-track/off-track achievements by program are detailed in Table 4. Among the respondents who reported reasons for being off-track (*N*=23), time (60%) and money (52%) were the most frequently reported reasons, followed by grades (40%).

**Table 3.** Primary and Secondary Outcome Achievements by Program (*N*=105)

Program	SUMP (N*=61), *n (%)	Monte HOP (N*=18), *n (%)	DSSROP (N*=26), *n (%)	Total respondents (N*=105), *n (%)
Achieved primary outcome	19 (31)	3 (17)	12 (46)	34 (32)
Achieved secondary outcome	7 (12)	4 (22)	5 (19)	16 (15)
Did not achieve primary or secondary outcome	35 (57)	11 (61)	9 (35)	55 (52)

**Table 4.** On Track/Off-Track by Program

Outcome	SUMP (N*=84), *n (%)	Monte HOP (N*=34), *n (%)	DSSROP (N*=29), *n (%)	All programs (N*=147), *n (%)
On track	61 (73)	24 (71)	22 (76)	107 (73)
Off track	23 (27)	10 (30)	7 (24)	40 (27)

For the first open-ended question, participants (*N*=133) identified distinct aspects of the programs as most valuable, resulting in 31 distinct codes. In descending order of frequency, the students identified the overall most valuable components to be (1) clinical exposure (40 mentions); (2) mentorship (25 mentions); (3) career exposure (20 mentions); and (4) research opportunities (16 mentions). Analysis of the remaining two open-ended questions resulted in three themes demonstrating the influence of the pipeline programs on participant's academic and career trajectories.

### Theme 1: Dreams realized

For many students in the programs, dreams of working in health care became attainable. Often, the experience gave them the courage to pursue their passions. “Without a program that specifically caters to this [underserved] population, we run the risk of not achieving our educational potential- or not even knowing it is there.” Several participants are the first in their families to attend college and did not have the role models within their social circles to inspire and guide them toward the health professions.

Their confidence was enhanced. “Before, I used to think that attending medical school was nearly impossible, but after SUMP, I feel so prepared to pursue my goal of becoming a doctor.” If such programs were not offered, participants identified the potential loss of talent and dreams unfulfilled with personal- and society-level consequences.

Not having a program would be absolutely devastating to students from underrepresented minority and economically disadvantaged backgrounds. I have been discouraged by both academic administration as well as healthcare professionals. I have been encouraged to choose another career goal based on my ethnicity, financial status, and gender.

For some, the program offered the necessary resources, networks, and encouragement that were not available on their college campuses.

In addition, they valued their pipeline program's goals of increasing the diversification of the health care workforce because of the impact that their own dreams being realized could have on the communities with whom they identify. “It is important to have providers native to not only the language but to the culture as well to provide comfort to a patient.” Racial, ethnic, and/or language minority patient populations are also affected if there were decreased opportunities to participate in pipeline programs.

### Theme 2: Professional identity formation

Participants were inspired to take greater initiative and persevere despite the obstacles in their path. Participants believed in themselves more and realized they could accomplish their goals. Their will was fortified and their resilience increased.

Sometimes I felt like I wasn't as strong of an applicant as many of the other people that I knew were applying for graduate/medical school. Participating in this program showed me that I could perform at the same level, or even better, and gave me the confidence to go through with applying to medical school.

Participants found the programs to be enriching experiences, as they gained confidence, focus, and clarity. They became more confident in embracing their professional identity along with peers, faculty mentors, and role models.

Meeting other URM professionals allowed students to visualize their own career paths and form a professional identity. They realized that they could be advocates for patients, their communities, and other students. Participants also discovered career options serving underserved communities. “I was exposed to strong female minority doctors who served as strong role models for me. They were in primary care and served minority and underserved communities, which I am now pursuing.” Participants valued learning about health disparities, social determinants of health, and issues around access to health care. Many were motivated to incorporate these topics into their future careers. Programs and mentors fostered or enhanced a desire to work in underserved communities through lectures and shadowing.

Many relished their relationships with medical students and practicing physicians and scientists. It often helped to see role models who come from similar backgrounds and learn how they overcame obstacles to achieve their goals. These examples encouraged and motivated them to persevere and realize they, too, could achieve their goals.

We need to see individuals with our backgrounds that have made it in the sciences because that gives us the confidence to achieve. Pipeline programs are important to making the necessary connection between young people from underrepresented groups interested in the sciences and underrepresented professionals.

As a consequence of being immersed in a summer program with a peer network of high-achieving students with similar goals, participants' perspectives of the career possibilities within the medical field were broadened. Several participants felt that they were the only URMs on their campuses undertaking the medical school application process. “It's not just being in the program, but having colleagues beyond our institutions striving for the same goal. When premed friends in our immediate vicinity are changing paths, we have each other to touch base with and encourage upward.” Meeting other ambitious URM students helped overcome feelings of isolation and expanded their network of peers with similar personal and emerging professional identities. Their relationships lasted longitudinally, not just with their peers, but with many of the faculty and their mentors as well.

Faculty level connections often resulted in timely introductions to new career opportunities, letters of recommendation, feedback on personal statements, and often intangible, but no less important benefits. “By allowing me to interact directly with physicians in the field, the program motivated me immensely to work harder in my academic and personal pursuits so that I may reach my goals one day and work alongside the physician mentors I developed close relationships with.”

They believed the relationships they built with both their peers and mentors helped them clearly see the pathway to an advanced degree in health care. They perceived that their desired professional identity became more and attainable. “The program is structured with the awareness that the students in the program have had little exposure to the field. It provides an infrastructure to gain exposure and advice that is often understated [or unavailable].” Before their experience in these programs, many participants perceived the process of getting into medical or graduate school as a mystery only known to “others” and had limited opportunities to find suitable mentorship. Meeting and having access to peers, medical/graduate students, and practicing health professionals and scientists with similar personal identities demystified the “black box” of getting into medical or graduate school.

### Theme 3: Addressing systemic inequities

Several participants believed that such programs help level the playing field for all applicants and increase equity of opportunity to enter a career in science or the health professions. Some noted that many of their college classmates had parents in the medical field who provided them with guidance and insight.

Without these pipeline programs, navigating the path to a career in medicine will be difficult for many underrepresented groups who are often not privileged to attend [prestigious] schools or know people who can provide pertinent information for achieving their goals.

Without pipeline programs, the cycle of privilege could be perpetuated, because the students served by pipeline programs often do not have the opportunities to become competitive applicants at their colleges or the necessary connections within their networks.

Participants identified many tangible benefits from the programs that helped them overcome their real and perceived systemic, economic, and educational disadvantages. The research components enhanced their skills and confidence, also allowing for continued and new experiences beyond participation in the program, even leading to a first publication for some. “Undertaking the research endeavor was initially overwhelming, but after working with my principal investigator and the graduate students in the laboratory, I learned how to design my own experiments, and put together a scientific poster.” Study skills, time management, organizational strategies, and balancing a challenging work load with their personal responsibilities were also cited by several participants. “I distinctly remember one of the lectures was about time management and how to effectively study for exams. I now use those skills for my academics. The program taught me how to manage time and academic life.” Participants felt empowered with a road map to navigate the application process, finance their education, and maximize their competitiveness for ultimate matriculation to medical or graduate school.

## Discussion

The results of our quantitative analysis indicate that 73% of our respondents matriculated into MD or PhD programs, masters-degree level health professions program, or were on track to achieve these outcomes. Close to three quarters (74%) of our respondents identified as black or Hispanic/Latino/a/x. Our outcomes suggest that our programs have a significant positive impact on the academic and career trajectories of URM students. Our programs successfully prepared participants for medical and graduate school matriculation and fortified their resilience, motivating them to persist along their chosen path. Participants valued exposure to clinical and research environments, as well as learning about career options and receiving direct mentorship. Participation enabled them to pursue their dreams, see themselves in others with a similar professional identity, and mitigate the impact systemic inequities had on their individual trajectories.

In 2003, Grumbach et al. wrote in their review of 19 controlled studies “the most rigorously designed studies indicated that special summer enrichment programs can boost the success of URM… students in applying to medical school by approximately 25%”^9^ (p. 79). Our findings are in line with similar undergraduate pipeline programs serving URM and disadvantaged students. For instance, one similar program reports 69% (27/39) of graduates matriculated into or on-track to enter health professions graduate programs^12^; another pipeline program's longitudinal 10-year retrospective study revealed that 74% of graduates matriculated into graduate school in the biomedical sciences.^16^ Our study presents a unique approach to program evaluation of pipeline programs by applying a cross-sectional, long-term design to collect participants' educational and career outcomes and their hindsight perspectives of the factors which influenced these outcomes.

Our qualitative analysis revealed that students valued components of the program that provided direct contact and insight into the health care profession that they would not have otherwise had the opportunity to pursue with. Participants also valued the long-term relationships they formed and opportunities to work with and learn from URM mentors, faculty, and peers. These findings align with SCCT, in that, learning experiences within the pipeline programs which increased self-efficacy and favorable outcome expectations, in turn, influenced career interests, goal choices, and intentions to pursue a health career path.^21^ In addition, contextual supports in the form of mentors, peers, and academic resources, as well as barriers, including finances and grades, both directly and indirectly influenced career interests, choice goals, and subsequent career attainment, as evidenced by reasons for being off-track.^20^ In addition to the most frequently cited impactful components of the programs, our themes have implications for the design of future pipeline programs and the prioritization of components in an environment with limited resources and funding opportunities.

One implication involves career choice and professional identity formation. SCCT holds that self-efficacy and outcome expectations developed through social learning experiences increase one's motivation to persist toward academic and career goals.^21^ Mentorship from URM faculty members, and its positive effects on URM professional identity formation, would therefore be a key component of a successful pipeline program. URM faculty members represent only 8% of faculty in US medical schools.^24^ They are often tasked with many roles beyond mentoring of URM students and trainees, colloquially called the “minority tax.”^25,26^ The importance of mentorship combined with the difficulty URM and women have in obtaining suitable mentors,^25^ implies that pipeline programs might benefit from incorporating willing faculty from all racial, ethnic, and gender backgrounds to serve as mentors for URM students.^27^ Students can still be exposed to role models from similar racial, ethnic, and socioeconomic backgrounds during their clinical experiences, through didactics, and potentially with more time-limited programs such as a “Meet the Professor” format. Creative solutions to expose students to successful role models of similar backgrounds while having the more intense relationship with a mentor of any background could still capitalize on the power of seeing role models in positions to which they aspire, as SCCT predicts.

In addition to the implications for the individual students, our findings have societal implications. A more racially and ethnically diverse class benefits all students.^21,,22^ A diverse health care workforce enhances patient outcomes and decreases health disparities.^3^ URM medical students are more likely to work in minority and underserved patient populations.^3,28^ Successful pipeline programs would therefore not only benefit individual students, but have potential to benefit communities and the health of our society as a whole.^3,29^

One final implication of our results could inform recruitment approaches taken by pipeline programs. Although we report the outcomes of how many students achieved or were on track to obtain a medical degree, further research is needed as to what percentage of students entering medical school would reflect “success” of a program. Setting a percentage benchmark arbitrarily may risk increased selection of students who would reach medical school regardless of participation in a pipeline program and whose lives would not be markedly affected by program participation. We advocate for a holistic approach and flexibility to allow programs to recruit students who have the potential and interest to matriculate into health professions schools and who need the “intangible” supports as evidenced by our qualitative results. We recognize this may lead to an overall decrease in the percentage of “successful” participants, but will likely lead to an increase in medical school matriculants from disadvantaged backgrounds and enhance overall diversification of the health care workforce in line with the social justice mission of many of these programs.^12,13,16^

### Limitations

This evaluation is limited due to the lack of information about the students' baseline characteristics before participating in the program, as well as our inability to control for impactful events or experiences between the time of program participation and this follow-up evaluation. Given the existing evidence,^9–17^ a randomized controlled trial design may have been considered unethical unless we were able to offer an alternative intervention program to a control group; this was not feasible with our limited resources. Inactive email addresses limited our ability to reach many participants. The disproportionate representation of SUMP participants (57%) among respondents is likely because the SUMP program's administrator (H.S.) used a personal Facebook page to remain in contact with program alumni over time. Unlike the other two programs, SUMP also used an electronic application process, which allowed for contact data to be more easily exported. We were unable to ascertain if our sample was representative of URM students who participate in educational pipeline programs because, to our knowledge, those data are not published. Response bias may have led successful participants to disproportionately respond or others to respond with more socially desirable outcomes. However, the authors have learned through nonsystematic follow-up that many nonresponders actually entered medical or graduate school and, thus, became too busy to respond. Our findings illustrate the challenges to long-term follow-up and the need for funding to support broader data-sharing agreements.

## Conclusions

Our pipeline program served to enhance students' self-efficacy and resulted in successful academic and career trajectories to become health professionals at the doctorate and master's degree level. Beyond the skills and academic preparation offered, the programs offered support both peer support and mentorship, to facilitate success. Given the importance of these supports on the formation of their professional identity, pipeline programs should take a holistic approach considering the existing networks of applicants for participation. Our findings can inform future research to develop outcome metrics that take these intangibles into consideration. Returning to the federal funding levels of 15 years ago could more than double the scale of the Health Career Opportunity Program and support long-term program evaluations so as to develop efficient, effective best practices to diversify the health care workforce and improve the health of all communities.

## Abbreviations Used

SCCT: Social Cognitive Career Theory
SUMP: Summer Undergraduate Mentorship Program
URM: underrepresented minorities
